# Stimuli-Sensitive Hydrogel Based on N-Isopropylacrylamide and Itaconic Acid for Entrapment and Controlled Release of *Candida rugosa* Lipase under Mild Conditions

**DOI:** 10.1155/2014/364930

**Published:** 2014-05-25

**Authors:** Nikola Milašinović, Zorica Knežević-Jugović, Nedeljko Milosavljević, Marija Lučić Škorić, Jovanka Filipović, Melina Kalagasidis Krušić

**Affiliations:** ^1^Department of Forensics, The Academy of Criminalistic and Police Studies, Cara Dušana 196, 11080 Belgrade, Serbia; ^2^Department of Biochemical Engineering and Biotechnology, Faculty of Technology and Metallurgy, University of Belgrade, Karnegijeva 4, 11000 Belgrade, Serbia; ^3^Department of Organic Chemical Technology, Faculty of Technology and Metallurgy, University of Belgrade, Karnegijeva 4, 11000 Belgrade, Serbia

## Abstract

Stimuli responsive pH- and temperature-sensitive hydrogel drug delivery systems, as those based on N-isopropylacrylamide (NiPAAm) and itaconic acid (IA), have been attracting much of the attention of the scientific community nowadays, especially in the field of drug release. By adjusting comonomer composition, the matrix is enabled to protect the incorporated protein in the highly acidic environment of upper gastrointestinal tract and deliver it in the neutral or slightly basic region of the lower intestine. The protein/poly(NiPAAm-co-IA) hydrogels were synthetized by free radical crosslinking copolymerization and were characterized concerning their swelling capability, mechanical properties, and morphology. The pore structure and sizes up to 1.90 nm allowed good entrapment of lipase molecules. Model protein, lipase from *Candida rugosa,* was entrapped within hydrogels upon mild conditions that provided its protection from harmful environmental influences. The efficiency of the lipase entrapment reached 96.7%, and was dependent on the initial concentration of lipase solution. The swelling of the obtained hydrogels in simulated pH and temperature of gastrointestinal tract, the lipase entrapment efficiency, and its release profiles from hydrogels were investigated as well.

## 1. Introduction

Hydrogels have attracted particular attentions since they can undergo abrupt changes between their collapsed and swollen states in response to various environmental stimuli changes [1–4]. Hydrophilic hydrogels exhibit unique well-defined physicochemical properties and reproducible drug release profiles [5] that make them advantageous for biomedical applications including drug delivery [6]. The use of such hydrogels in drug delivery systems is particularly important as they are often applied to protect the protein from hostile conditions such as low pH in the stomach [7] since the therapeutic application of hydrophilic macromolecules is associated with some serious problems, especially upon their oral administration [2]. A successful controlled release device depends on technological factors such as protein-loading efficiency, protein integrity, and desired release characteristics, such as the release of protein in the exact position or the desired site in the body [8].

The increased interest for the biotechnological production of itaconic acid [9, 10] and the fact that it is obtained from nonpetrochemical resources [11] led to an increase of its utilization in the synthesis of temperature-sensitive hydrogels [12, 13] that exhibit pH sensitivity as well [14–17], since itaconic acid facilitated the tuning of the release of model protein from the produced temperature- and pH-sensitive systems. For successful entrapment, the hydrogel must have the mesh size large enough to allow molecules of the substrate and of the reaction product to diffuse in and out and to keep the enzyme entrapped in the hydrogel support. The network parameters and hydrogel morphological and mechanical properties can be easily controlled by appropriate selection of reaction mixture composition and reaction conditions [18–20]. One of the biggest challenges for researchers nowadays is the oral application of the protein, limited due to the enzyme inactivation in the gastrointestinal tract and low permeability of these substances. However, using the corresponding hydrogels, these problems could be overcome.

In this work, we developed such pH- and temperature-sensitive hydrogel drug delivery system, based on N-isopropylacrylamide (NiPAAm) and itaconic acid (IA), which was able to protect the incorporated model protein in the highly acidic environment of upper GI tract and deliver it in the neutral or slightly basic region of the lower intestine. Therefore, the purpose of this study was to prepare the protein/poly(NiPAAm-co-IA) hydrogels and investigate its possibility to be applied as a protein controlled release device. Model protein, lipase from* Candida rugosa* (CRL), was entrapped within hydrogels upon mild conditions that provide its protection from harmful environmental influences. The structure and properties of the hydrogels were characterized, and the release behavior and release mechanism of CRL from the hydrogels were also investigated in detail.

## 2. Materials and Methods

### 2.1. Materials

Both monomers, itaconic acid (IA) and N-isopropylacrylamide (NiPAAm), were purchased from Acros Organics (Belgium). N,N′-methylenebisacrylamide (MBA) from Serva Feinbiochemica (Germany) was used as the crosslinking agent. The initiator and accelerator, potassium persulfate (PPS) and potassium pyrosulfate (PPyroS), were obtained from Merck & Co., Inc. (Germany), and Acros, respectively. Lipase from* Candida rugosa* (CRL) was kindly obtained from Sigma-Aldrich Chemie Gmbh (Germany). NiPAAm was recrystallized from benzene/*n*-hexane mixture (35/75) before its utilization. Other materials were used as received, without purification. Different pH value aqueous media were prepared using hydrochloric acid (LaChema, Czech Republic), potassium chloride (Zorka Šabac, Serbia), sodium dihydrogen phosphate dihydrate, and disodium hydrogen phosphate dodecahydrate (Lach-Ner, s.r.o., Czech Republic). Molar concentrations of all buffer solutions used were 0.2 M. Distilled water was used for all syntheses and the preparation of the buffer solutions.

### 2.2. Synthesis of the Hydrogels

The syntheses of hydrogels of various compositions were performed by free radical crosslinking copolymerization at 25°C in the nitrogen atmosphere, according to the modified procedure, reported previously [17]. Briefly, NiPAAm and IA were separately dissolved in water. PPS and PPyroS as redox couple in an amount of 1.0 wt.% with respect to monomers were added to the IA solution prior to polymerization. The concentration of the crosslinking agent, MBA, was 2.0 and 4.0 wt.% with respect to monomers. After being purged by the nitrogen, the reaction mixture was poured between two glass plates (21 × 6 × 0.4) cm, sealed with a PVC spacer (0.3 cm thick).

The reaction time depended on the concentration of the crosslinking agent, as well as on the NiPAAm/IA comonomer weight ratio in the initial mixture and was in the range of 12 to 48 h. After the reaction was completed, the gels were cut into discs and immersed in water which was changed daily for a week, to remove unreacted reactants. The discs were dried at room temperature for a day and then at the temperature of 37°C to xerogels (0.10 ± 0.01 cm thick and 0.70 ± 0.10 cm in diameter). The PNiPAAm hydrogel has been synthetized under the same conditions as used for the copolymers with 2.0 wt.% and 4.0 wt.% of MBA. The samples were labeled as NiPAAm/IA/MBA 85/15/2, 90/10/2, 95/5/2, and 100/0/2 and 85/15/4, 90/10/4, 95/5/4, and 100/0/4. The first two numbers correspond to the comonomer NiPAAm/IA weight ratio, and the third one corresponds to the concentration of the crosslinking agent, MBA.

### 2.3. Entrapment of Lipase from* Candida rugosa*

In order to entrap a model protein into the system, xerogels were immersed in enzyme solutions of different temperatures, pH values, and CRL concentrations, as a common method for drug entrapment in hydrogels [19]. The effect of various factors on the lipase activity after entrapment was presented in Supplementary Material (Tables  1–3) available online at http://dx.doi.org/10.1155/2014/364930. The optimal time for CRL entrapment was determined to be 3 h. Protein concentrations in solutions before and after entrapment, as well as in washing solutions, were evaluated by the Lowry method [21]. The protein calibration curve was constructed using CRL solutions with known enzyme concentration. Loading capacity (*P*_*g*_, mg/g_xerogel_) was determined from the following equation:

(1) $$\begin{array}{c} P_{g}=\frac{\left[C_{0}V_{0}-\left(C_{1}V_{1}+C_{2}V_{2}\right)\right]}{w}, \end{array}$$

where *C*_0_ is the protein concentration of the initial enzyme solution (mg/mL) and *V*_0_ its volume (mL), *C*_1_ is the protein concentration in the solution after entrapment (mg/mL) and *V*_1_ its volume (mL), *C*_2_ is the protein concentration of the washing solution (mg/mL) and *V*_2_ its volume (mL), and *w* is the weight of xerogels used (g).

The efficiency of CRL entrapment (EE) into hydrogels was calculated using the following equation:

(2) $$\begin{array}{c} \text{EE}\left(\%\right)=\frac{\left(\text{the}\;\;\text{amount}\;\;\text{of}\;\;\text{entrapped}\;\;\text{protein}\right)}{\left(\text{the}\;\;\text{initial}\;\;\text{amount}\;\;\text{of}\;\;\text{protein}\right)}\times 100. \end{array}$$

### 2.4. Swelling Degree Studies

The swelling degree of the hydrogels at proper time intervals was monitored gravimetrically, by immersing xerogel discs in buffer solutions of 2.20 ± 0.01 and 6.80 ± 0.01 pH values in order to simulate the gastric and intestinal pH at 37°C. The swelling degree was calculated using the following equation [6]:

(3) $$\begin{array}{c} q=\frac{W_{t}}{W_{0}}, \end{array}$$

where *W*_0_ represents the weight of the xerogel disc and *W*_*t*_ is the weight of the swollen hydrogel at specific time *t*.

### 2.5. Mechanical Properties

For the strain-frequency sweeps tests hydrogel discs swollen to equilibrium were used. The measurements were performed using a* Rheometrics 605* Mechanical Spectrometer, with parallel plate geometry (25 mm in diameter). The sizes of discs were 25 mm in diameter in order to fit the parallel plate diameter. The gap for the samples was about 2.55 mm (with the variation of less than 10% for all tested samples). The complex shear moduli were measured as a function of angular velocity (*ω*), from 0.1 to 100 rad·s^−1^, at 37°C. The strain applied was 20%.

### 2.6. Morphology of the Hydrogels

The morphology measurements of the hydrogel samples were conducted using* JEOL JSM-5800* Scanning Electron Microscope. Prior to the SEM morphological experiments the hydrogel samples were lyophilized, frozen in liquid nitrogen, and then broken in order to avoid the deformations caused by the fracture of the samples. Later on, the samples were coated with platinum under vacuum using* Polaron SC502* sputter coater.

### 2.7. Protein Release Study from the Hydrogels

To study the effect of the hydrogel composition, comonomer and crosslinking agent concentration, on protein release profiles the* in vitro* release experiments were performed at 37°C in the buffer solution that simulated pH of biological fluids. Briefly, all hydrogel samples were immersed in a beaker filled with 10 mL buffer solution of a targeted pH value. Samples were first placed into beaker filled with solutions of pH 2.20 ± 0.01 for two hours and then transferred into beaker filled with buffer solutions of pH 6.80 ± 0.01 (to completion of one week). To simulate the gut movement the samples were placed in a shaking incubator with a mild shaking motion (50 rpm) during the studies. The average volume of the xerogels prior to the release kinetic studies was 0.0343 ± 0.0033 mL. The loaded hydrogels were stored in the dry state before the release experiments.

At proper intervals, 2 mL of solution was taken for released protein measurement and returned back to the beaker to maintain the same condition throughout the experiment. Both sterile and sink conditions have been maintained throughout the experiments. The samples were analyzed spectrophotometrically at 225 nm by using bovine serum albumin as a standard (Ultrospec 3300* pro *UV/Visible Spectrophotometer, Biochrom Ltd.). The enzyme concentration was determined from a standard curve which is constructed for each measurement. Mean and standard deviation of the results from three independent experiments were calculated using Microsoft Excel (Redmond, WA, USA) software. All data were reproducible within ±5% of accuracy. Finally, the results of release study were presented in terms of cumulative release as a function of time. The enzymatic activity of samples was also analyzed using the Sigma lipase activity method as previously described [21].

## 3. Results and Discussion

### 3.1. FT-IR Analysis

**Figure 1:**
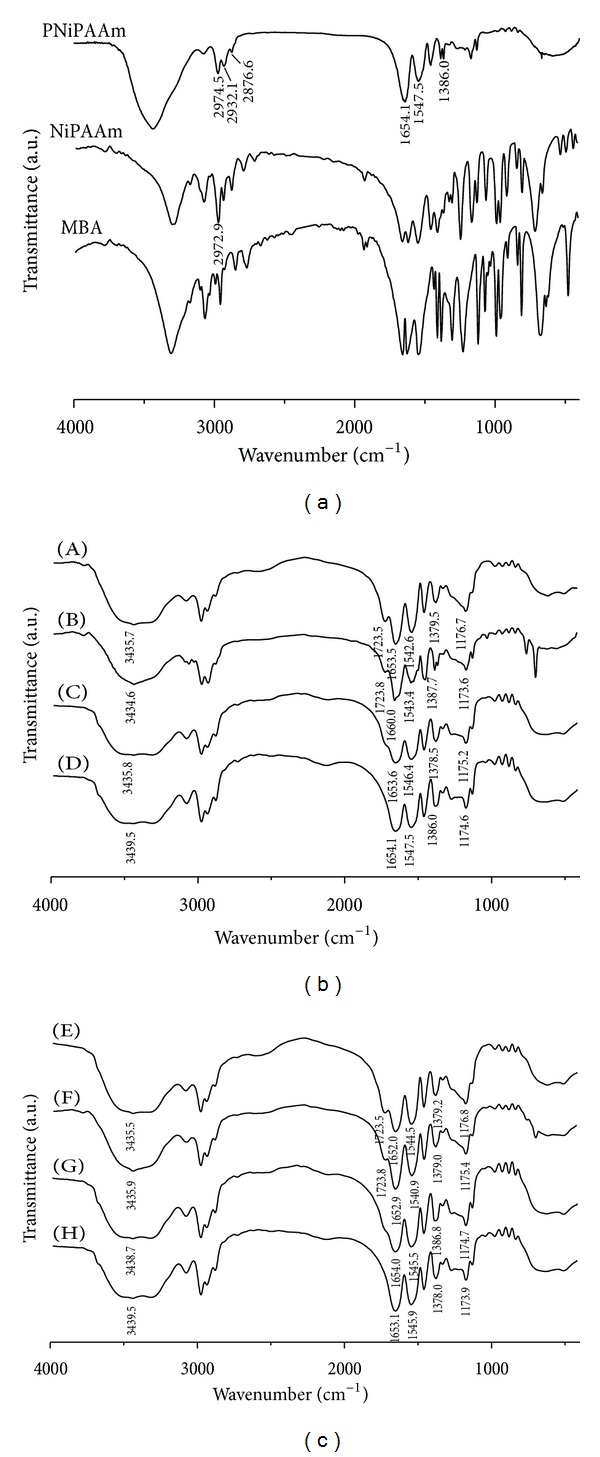
FT-IR spectra of hydrogels: (a) spectra of PNiPAAm, NiPAAm, and MBA; (b) spectra of hydrogels with 2.0 wt.% of MBA ((A) 85/15/2; (B) 90/10/2; (C) 95/5/2; (D) 100/0/2); and (c) spectra of hydrogels with 4.0 wt.% of MBA ((E) 85/15/4; (F) 90/10/4; (G) 95/5/4; (H) 100/0/4). The first two numbers in the sample labels correspond to the comonomer NiPAAm/IA weight ratio, and the third one corresponds to the concentration of the crosslinking agent, MBA.

FT-IR spectra of the crosslinking agent, NiPAAm monomer, and PNiPAAm are presented in Figure 1(a). Figures 1(b) and 1(c) show FT-IR spectra of homo- and copolymer hydrogels of different composition, both monomer content and crosslinking agent concentration.

Both NiPAAm and MBA as acrylamide derivatives, as well as PNiPAAm, show similar spectra with characteristic bands. Namely, the band at 2974 cm^−1^ corresponds to the C–H asymmetric and symmetric vibrations and N–H stretching vibrations. The band at 2877 cm^−1^ are assigned to the C–H asymmetric and symmetric vibrations from isopropyl group. Additionally, amide bands I and II are evident at 1654 and 1548 cm^−1^. The vibration of C–H bond as well as the band of symmetric isopropyl group is visible at 1386 cm^−1^. Furthermore, FT-IR spectrum displays peak at 2932 cm^−1^ related to asymmetric behavior of CH_2_ group.

FT-IR spectra of hydrogels are similar. Each spectrum shows a wide band in the area of 3700–3100 cm^−1^ which corresponds to the O–H stretching vibration of carboxylic groups in itaconic acid and N–H stretching vibration of NiPAAm. Stretching of C–H group from NiPAAm is also noticeable at 2976 cm^−1^. Peak at 1723 cm^−1^ originates from the vibration of the carbonyl group in itaconic acid [22]. Typical amide I band and amide II band of NiPAAm appear around 1650 cm^−1^ and 1540 cm^−1^, respectively. Two typical bands of C–H vibrations of nearly the same intensity at 1386 and 1379 cm^−1^ correspond to the stretching vibration of C–H bond of CH (CH_3_)_2_ groups. The band around 1174 cm^−1^ originates from the amide III band in P(NiPAAm) [23]. Band at 1207 cm^−1^ corresponds to C–O stretching of carboxylic groups in itaconic acid. At 1400 cm^−1^ some C–O–H banding in plane is visible. Characteristic bands in the FT-IR spectra correspond to the absorption bands of hydrogels characteristic for homopolymers of poly(itaconic acid) and poly(N-isopropylacrylamide) but are slightly shifted in relation to the wavenumbers of pure polymers because of the crosslinking reaction and the formation of the hydrogel polymer network.

### 3.2. SEM Analysis

**Figure 2:**
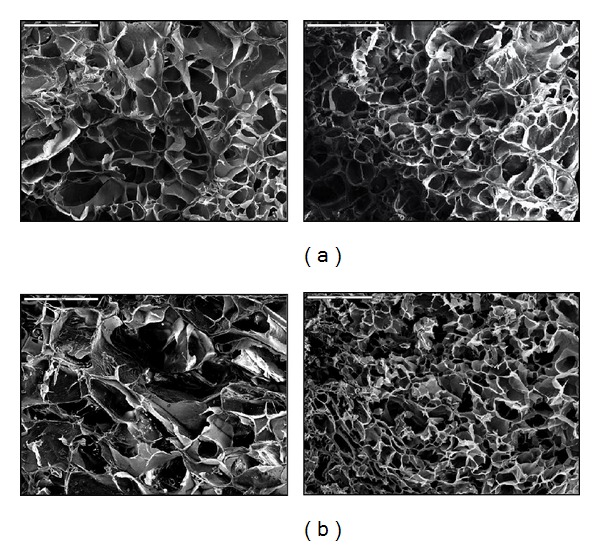
SEM micrographs of hydrogels: (a) 90/10/2 (left) and 90/10/4 (right); (b) 85/15/2 (left) and 100/0/2 (right) swollen to equilibrium at pH 6.80 ± 0.01 at 37°C (“*bar*” 500 *μ*m, 70x).

Figure 2 shows the morphology of the P(NiPAAm/IA) copolymer hydrogels without lipase. It is obvious that hydrogels have a porous structure and that the pore size depends on the composition of the hydrogel. The effect of the crosslinking agents is seen in Figure 2(a), showing the morphology of samples with the same comonomers composition and indicating that the pore size is smaller when higher concentration of the crosslinking agent was used in hydrogel synthesis. Also, it is clear that addition of itaconic acid influences the pore size of the synthetized hydrogels since larger pores were observed for samples containing higher content of acid (15% and 0% of IA) and the same crosslinking agent amount (Figure 2(b)).

By applying the theory of the equilibrium swelling the determined pore sizes were in the range between 0.17 and 1.90 nm, when hydrogels were swollen to equilibrium in buffer solution of pH 6.80 ± 0.01 at 37°C.

### 3.3. DMA Analysis

**Figure 3:**
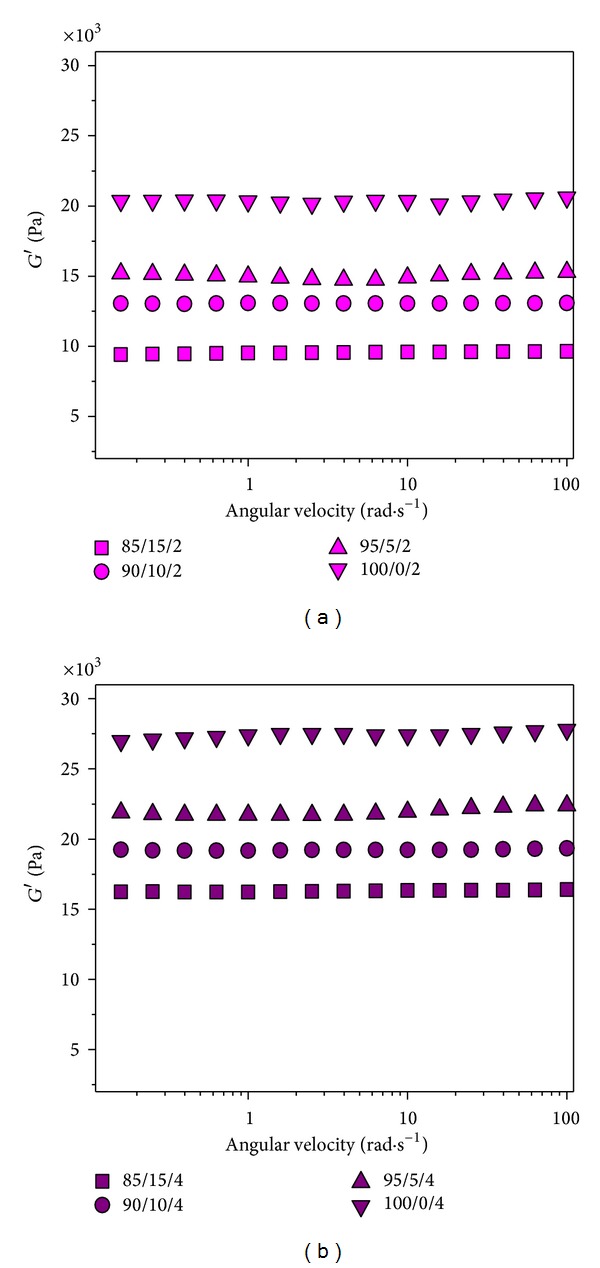
The shear storage moduli, *G*′, as a function of the angular velocity of P(NiPAAm/IA) hydrogels of different composition with (a) 2.0 wt.% and (b) 4.0 wt.% of the crosslinking agent swollen to equilibrium in pH 6.80 ± 0.01 at 37°C.

Mechanical properties, specifically the shear storage moduli (*G*′) as a function of angular velocity, of hydrogels samples with no CRL entrapped into them, are shown in Figure 3. The measurements were performed at 37°C on hydrogels swollen to equilibrium in buffer of pH 6.80 ± 0.01. As the objective was to examine the behavior of these hydrogels under conditions prevailing in the lower parts of GI tract. Also, at pH value of 6.80 both carboxylic acid groups are ionized, and the swelling behavior of hydrogels having IA is more pronounced, which affects the mechanical properties of the synthetized hydrogels. From these results it was determined that the comonomers ratio and the concentration of crosslinking agent affected the mechanical properties of these hydrogels. As expected, with an increase in the degree of crosslinking, more dense network is formed causing stronger mechanical properties, but the absorption of the surrounding fluid and hydrogel swelling is reduced.

With increasing the content of the degree of crosslinking agent (from 2.0 to 4.0 wt.%) the mechanical properties of hydrogels are improving, as is clearly seen from the results in Figures 3(a) and 3(b). The shear storage moduli decrease with increasing the content of itaconic acid in the initial reaction mixture, giving weaker mechanical properties to the synthetized hydrogels. Higher shear storage moduli were obtained with hydrogels that swell less.

From Figure 3 it can be concluded that the influence of the crosslinking agent and itaconic acid content is quite distinct. The largest shear storage moduli have sample which does not contain itaconic acid and the smallest sample with the highest content of itaconic acid, which is consistent with the results of the swelling (data not shown). The shear storage moduli, *G*′, is independent of the angular velocity for all samples.

### 3.4. The Loading Capacity

The loading capacity during the entrapment of lipase was in the range between 74.7 and 365.7 mg/g_xerogel_, depending on the initial concentration of the CRL solution and concentration of the crosslinking agent, MBA. As the concentration of the lipase in the initial solution increases from 0.2 to 1.0 mg_enz_/mL the amount of the CRL entrapped into hydrogels initially rapidly increases and then reaches a maximum value of 365.7 mg/g and 264.3 mg/g, for xerogels with 2.0 and 4.0 wt.% of crosslinking agent, respectively. The observed behavior may be a result of the lipase surface saturation, since lipase during the entrapment process is preferably binding to the hydrogel surface [24–30]. Furthermore, some diffusion limitations due to the lipase molecule size exist, thus hindering lipase penetration within the polymeric matrix (to a greater extent) during the swelling process. In addition, the binding of lipase to the surfaces of the hydrogel matrices facilitates its rinsing from the carrier.

The polymerization time for synthetized samples was 48 h for the sample with 2 wt.% of MBA and 24 h for a sample with a 4 wt.% of MBA. Samples with no or very small amount of itaconic acid are good enough to be applied as carriers for CRL as model protein. Samples 95/5/2 and 95/5/4 were taken as representative and used for comparison reasons in order to test the entrapment efficiency and the controlled release of a lipase.

### 3.5. Hydrogel Swelling Behavior in Simulating Conditions of the Gastrointestinal Tract

**Figure 4:**
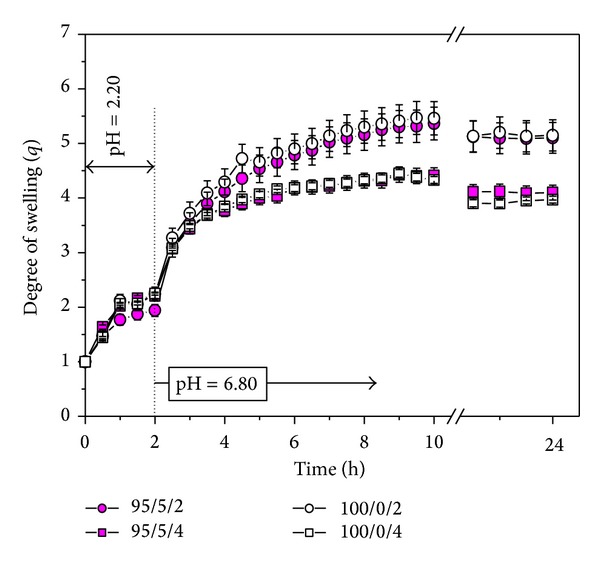
The dependence of the degree of swelling on time for 95/5/2, 95/5/4, 100/0/2, and 100/0/4 hydrogels swollen in solutions of 1 mg/mL of lipase concentration at 37°C simulating the pH of the gastrointestinal tract. Each data point is calculated based on three measurements (*n* = 3).

Considering the fact that the aim of this study was to investigate the potential application of these hydrogels as carriers in controlled release of the drug substance, tests are performed by simulating the pH and temperature in the GI tract. Swelling of different samples of P(NiPAAm/IA) hydrogels was monitored at 37°C in the solutions whose pH value was 2.20 ± 0.01 and 6.80 ± 0.01 simulating the pH in the stomach and in the lower GI tract (Figure 4). Swelling of the samples was carried out in such a way as samples were immersed in a buffer of pH 2.20 ± 0.01 in the course of 2 h and then transferred into a buffer of pH 6.80 ± 0.01 to swell for the next 22 h (so that the total time of swelling was 24 h). The objective was to simulate the total residence time of the protein in the GI tract. The kinetics of samples swelling was monitored at proper time intervals until they reached the equilibrium state.

### 3.6. The Release Kinetics

**Figure 5:**
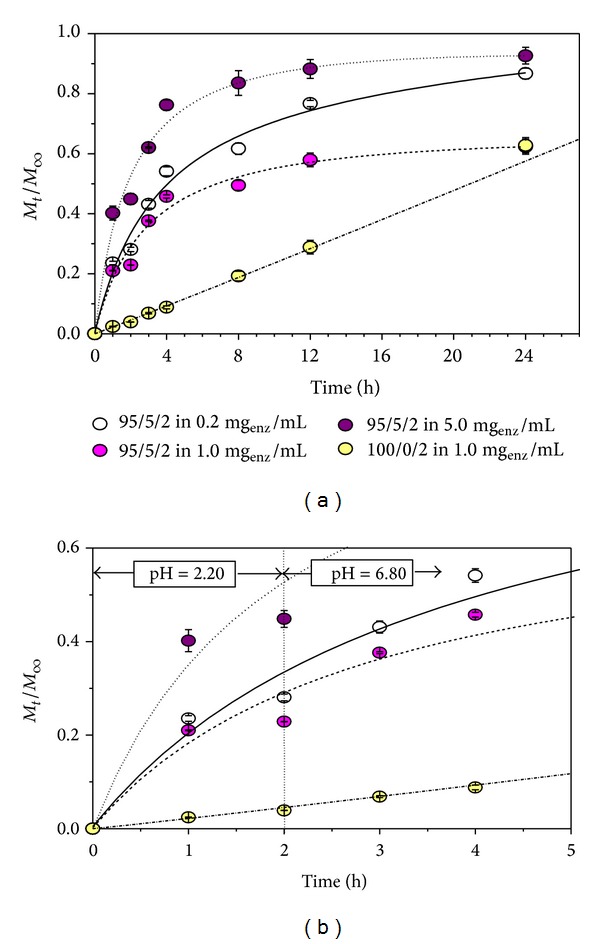
Mean normalized cumulative values of released lipase (*M*_*t*_/*M*_*∞*_) as a function of time at 37°C for hydrogels P(NiPAAm/IA) with 2.0 wt.% of the crosslinking agent. Each data point is calculated based on at least three measurements.

**Figure 6:**
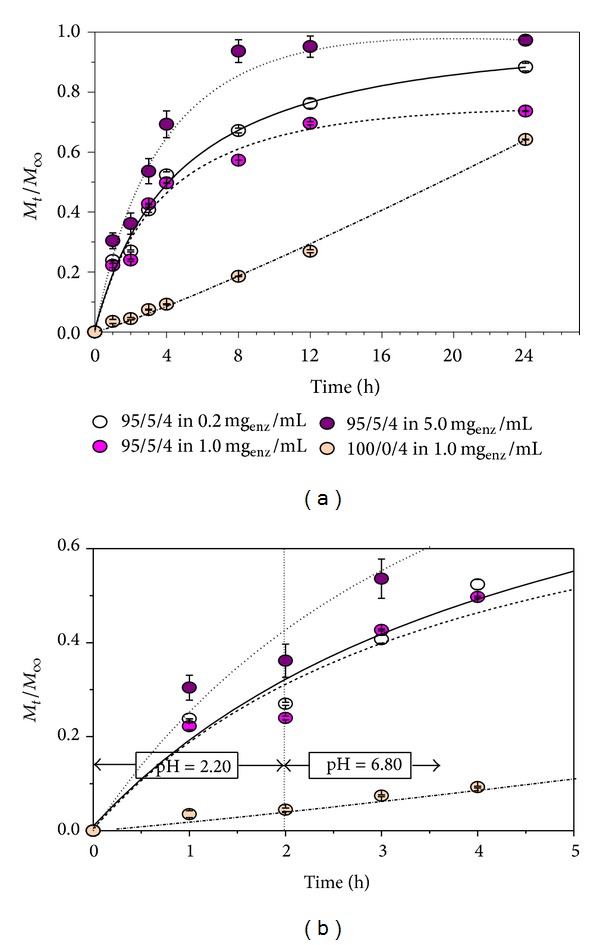
Mean normalized cumulative values of released lipase (*M*_*t*_/*M*_*∞*_) as a function of time at 37°C for hydrogels P(NiPAAm/IA) with 4.0 wt.% of the crosslinking agent. Each data point is calculated based on at least three measurements.

In order to investigate the potential of P(NiPAAm/IA) hydrogels as matrices for the controlled release of therapeutic proteins, the release of a lipase from hydrogel of different composition was monitored. The mean values of released lipase were presented with normalized curves and measurements' standard deviations, which were, for all tested samples less than ±5%, based on the average of at least three measurement values (Figures 5 and 6).

At a pH value of 2.20, sample 95/5/2 released from 22 to 55% of lipase, while sample 95/5/4 released from 25 to 35%. Much lower percentage of lipase was released from hydrogels with no itaconic acid (namely, 4.1% from sample 100/0/2 and only 4.4% from sample 100/0/4), offering controlled release of lipase, but also showing that these systems could be protein protective devices when applying them in the acidic surroundings, as such. When increasing the pH, the percentage of the released protein reaches almost maximum for the first 24 h (of 96 h in total) for the samples with even small amount of acid content.

For samples with a higher degree of crosslinking, less lipase from solution was bonded (Table 1). It is confirmed that hydrogels with lower content of MBA generally release model protein faster. Namely, samples that contain itaconic acid between 62 and 93% of entrapped lipase were released after 24 h from the samples with 2 wt.% of MBA, while between 73 and more than 97% of model protein was released from the hydrogel with 4 wt.% of MBA, which also confirms that the presence of the crosslinking agents affects the release rate of the protein. This percentage is much attenuated for the samples with no itaconic acid (58% from the hydrogel with 4 wt.% of MBA and 60% from the samples with 2 wt.% of MBA). The addition of crosslinking agent lessens the binding of proteins from the solution to the carriers, and the release of lipases at both investigated pH values is reduced (Figures 5 and 6).

As is the case, the biphasic release profile of lipase [31] was determined, confirmed by the initial phase of rapid release at pH 2.20 in the first 2 h, and then followed by a slower release at pH 6.80. Results of* in vitro* release kinetics show that small number of CRL was entrapped within the hydrogel matrices. The percentage of released lipase during 24 h was in the range between 58.2 and up to 97.4% for the samples that contain small amount of itaconic acid. The release kinetics of lipase was analyzed using Peppas mathematical model for the first 60% of the release process [32, 33]:

(4) $$\begin{array}{c} \frac{M_{t}}{M_{\infty }}=k\cdot t^{n}, \end{array}$$

where *M*_*t*_ is the total cumulative amount of released lipase at time *t*, *M*_*∞*_ represents the total cumulative amount of released lipase at swollen equilibrium state, *k* is a constant of swelling, and *n* represents the diffusion exponent that determines the transport mechanism [34]. When *n* has a value of 1, the release of the drug substance is independent of the concentration and time, corresponding to zero-order release kinetics. If *n* is less than 0.5 the diffusion is carried out according to the Fick's law, and if *n* is between 0.5 and 1, the release of the substance is affected by both diffusion and relaxation of the polymer.

By logarithmic transformation of (4) and applying the model of monitoring the first 60% of the lipase release process mentioned above [32] constants *k* and *n* are calculated and shown in Table 2, together with the half-time of the release process *M*_*t*_/*M*_*∞*_ = 0.5. Kinetic parameters for all samples that contain itaconic acid are not shown since their dependence of lipase released on time for the first 60% of the release process was not linear, so it was not possible to calculate these parameters by applying this model [32].

Furthermore, in this case, the hydrogels of lower content of the crosslinking agent exhibit higher lipase release rates as a result of decreased diffusion resistance with an increase in degree of swelling. Thus, *t*_1/2_ varies in the range from 1.8 to 6.6 h for hydrogels with itaconic content. This variable increases to 20.9 h for the samples with no itaconic acid. Additionally, it was concluded that the lipase release from these homopolymers closely follows the zero-order release kinetics [35] since they show no pH sensitivity. The results showed that the effect of the surrounding pH medium affected the release of lipase from hydrogels having incorporated acid component, but its affection is less pronounced than crosslinking agent content.

For practical application it is very important that the protein retains its biological activity after release process. Therefore, the released lipase activity after 96 h of releasing was determined by a standard olive oil emulsion method. It was found that the released lipase activity was only 1.2 IU/mg_enz_ (for 95/5/4 in 1.0 mg_enz_/mL) and 2.1 IU/mg_enz_ (for 95/5/2 in 5.0 mg_enz_/mL), probably due to the rapid release of the lipase and its rapid inactivation, and that more of its activity was preserved when lipase was released from hydrogels with no acid content since its release was sustained and less lipase was released in acidic medium. This suggests that this method with some modifications could potentially be used for the entrapment of model proteins of somewhat smaller size (and active components more resistant to acidic conditions) that are capable of diffusion inside the matrices under proposed mild conditions. In order to apply these systems for controlled release these modifications could be expressed primarily through reducing amount of acid from 5 wt.% in respect to monomers to some more gentle amounts.

## 4. Conclusion

In this paper the potential use of P(NiPAAm/IA) copolymer hydrogels as devices for the controlled release of lipase from* Candida rugosa* in lower parts of GI tract was investigated. The swelling of the samples in simulated pH and temperature of GI tract, the lipase entrapment efficiency, and its release profiles from hydrogels were investigated as well. It was found that, by adjusting the hydrogels composition (together with the degree of crosslinking), suitable release devices can be prepared.

The release of lipase was rapid from the hydrogels that contained itaconic acid and increased as the acid content increased. Future work will be focused on gentle reduction of acid content in order to improve model protein release profile. Adequate release profiles were achieved when homopolymers were used as matrices for the controlled release of lipase from* Candida rugosa*. Hence, the composition of P(NiPAAm/IA) hydrogels must be tuned to protect the drug substance during its stay in the acidic environment such as the stomach and to release it in the lower part of the GI tract.

## Supplementary Material

Tables 1-3 present the effect of temperature, pH of the buffer solution and lipase concentration on the lipase activity after entrapment.

## Figures and Tables

**Table 1:** The loading capacity and the entrapment efficacy for samples 95/5/2 and 95/5/4 swollen to equilibrium in solutions of different initial lipase concentrations (*C*).

*C*, mg/mL	95/5/2			95/5/4		
	*t*, h	*P*_*g*_, mg/g_xerogel_	*η*, %	*t*, h	*P*_*g*_, mg/g_xerogel_	*η*, %
0.2	48	97.9 ± 2.91	96.7	24	74.7 ± 1.73	95.0
1.0	48	337.8 ± 7.75	84.0	24	202.8 ± 4.29	43.0
5.0	48	335.6 ± 7.45	14.5	24	217.6 ± 4.53	11.1
10.0	48	321.0 ± 7.73	8.4	24	215.2 ± 4.70	6.1
20.0	48	365.7 ± 9.10	4.4	24	264.3 ± 5.66	3.1

*t*: polymerization time; *P*_*g*_: the loading capacity; *η*: entrapment efficiency.

**Table 2:** The lipase velocity release constant and mesh sizes for the hydrogels with no itaconic acid swollen to equilibrium in solution of lipase concentration of 1 mg/mL. Each data point is calculated based on at least three measurements.

Sample	pH = 2.20 ± 0.01 (2 h) → pH = 6.80 ± 0.01 (24 h) at 37°C		
	*k*, h^−1^	*n*	*t*_1/2_, h
95/5/2	—	—	6.82
100/0/2	0.022	1.02	20.86
95/5/4	—	—	4.05
100/0/4	0.018	1.05	19.28
