# Supplementary material for: Stimuli-Sensitive Hydrogel Based on N-Isopropylacrylamide and Itaconic Acid for Entrapment and Controlled Release of Candida rugosa Lipase under Mild Conditions
Source: Biomed Res Int. 2014 May 25;2014:364930. doi: 10.1155/2014/364930 (PMC4055544; doi:10.1155/2014/364930)
Supplement: Supplementary file 1 — Tables 1-3 present the effect of temperature, pH of the buffer solution and lipase concentration on the lipase activity after entrapment. [file 364930.f1.docx]

Supplementary Table 1. The effect of temperature on lipase activity after entrapment (xerogels swelling in lipase pH 7.00 ± 0.01 buffer solution of concentration of 1.0 mg_enz_/mL).

| ***Sample*** | ***t = 5 °C*** | | | | ***t = 25 °C*** | | | | ***t = 37 °C*** | | | |
| --- | --- | --- | --- | --- | --- | --- | --- | --- | --- | --- | --- | --- |
|  | ***IU/g_milled xerogel_*** | ***IU/mg_enz_*** | ***Y, %*** | ***IU/g_milled xerogel_*** | | ***IU/mg_enz_*** | ***Y, %*** | ***IU/g_milled xerogel_*** | | ***IU/mg_enz_*** | ***Y, %*** |  |
| 85/15/2/0 | 34.20 | 0.171 | 28.5 | 30.60 | | 0.153 | 25.5 | 21.20 | | 0.106 | 17.7 |  |
| 90/10/2/0 | 29.40 | 0.147 | 24.2 | 25.80 | | 0.129 | 21.5 | 10.60 | | 0.053 | 8.8 |  |
| 95/5/2/0 | 24.20 | 0.121 | 20.7 | 16.20 | | 0.081 | 13.5 | 5.20 | | 0.026 | 4.3 |  |
| 100/0/2/0 | 23.60 | 0.118 | 19.7 | 10.80 | | 0.054 | 9.0 | 2.00 | | 0.010 | 1.7 |  |
| 85/15/4/0 | 31.40 | 0.157 | 26.2 | 24.80 | | 0.124 | 20.7 | 15.60 | | 0.078 | 13.0 |  |
| 90/10/4/0 | 28.60 | 0.143 | 23.8 | 17.20 | | 0.086 | 14.3 | 8.20 | | 0.041 | 6.8 |  |
| 95/5/4/0 | 21.20 | 0.106 | 17.7 | 10.80 | | 0.054 | 9.0 | 3.60 | | 0.018 | 3.0 |  |
| 100/04/0 | 9.80 | 0.049 | 8.2 | 5.40 | | 0.027 | 4.5 | 0.60 | | 0.003 | 0.5 |  |

Supplementary Table 2. The effect of buffer solution pH on the activity of entrapped lipase (xerogels swelling was performed at 5 ºC in lipase pH 7.00 ± 0.01 buffer solution of concentration of 1.0 mg_enz_/mL).

| ***pH*** | ***Sample*** | ***IU/g_milled xerogel_*** | ***IU/mg_enz_*** | ***Y, %*** |
| --- | --- | --- | --- | --- |
| 6.04 | 90/10/2/0 | 13.60 | 0.068 | 11.3 |
|  | 95/5/2/0 | 6.40 | 0.032 | 5.3 |
|  | 95/5/4/0 | 3.40 | 0.017 | 2.8 |
| 7.00 | 90/10/2/0 | 29.00 | 0.145 | 24.2 |
|  | 95/5/2/0 | 24.80 | 0.124 | 20.7 |
|  | 95/5/4/0 | 21.40 | 0.107 | 17.8 |
| 8.00 | 90/10/2/0 | 22.00 | 0.110 | 18.3 |
|  | 95/5/2/0 | 20.00 | 0.100 | 16.7 |
|  | 95/5/4/0 | 8.60 | 0.043 | 7.2 |
| 8.99 | 90/10/2/0 | / | / | < 1.0 |
|  | 95/5/2/0 | / | / | < 1.0 |
|  | 95/5/4/0 | / | / | < 1.0 |

Supplementary Table 3. The effect of lipase concentration in the solution on the entrapped lipase activity (xerogels swelling was performed at 5 ºC and in a CRL solution of pH 7.00 ± 0.01).

| ***CRL solution concentration, mg/mL*** | ***Sample*** | ***IU/g_milled xerogel_*** | ***IU/mg_enz_*** | ***Y, %*** |
| --- | --- | --- | --- | --- |
| 0.2 | 90/10/2/0 | 12.20 | 0.061 | 10.2 |
|  | 95/5/2/0 | 10.80 | 0.054 | 9.0 |
|  | 95/5/4/0 | 5.80 | 0.029 | 4.8 |
| 1.0 | 90/10/2/0 | 29.00 | 0.145 | 24.2 |
|  | 95/5/2/0 | 24.80 | 0.124 | 20.7 |
|  | 95/5/4/0 | 21.40 | 0.107 | 17.8 |
| 5.0 | 90/10/2/0 | 63.20 | 0.316 | 52.7 |
|  | 95/5/2/0 | 39.60 | 0.198 | 33.0 |
|  | 95/5/4/0 | 27.80 | 0.139 | 23.2 |
| 10.0 | 90/10/2/0 | 65.00 | 0.325 | 54.2 |
|  | 95/5/2/0 | 46.00 | 0.230 | 38.3 |
|  | 95/5/4/0 | 39.20 | 0.196 | 32.7 |
| 20.0 | 90/10/2/0 | 62.80 | 0.314 | 52.3 |
|  | 95/5/2/0 | 45.40 | 0.227 | 37.8 |
|  | 95/5/4/0 | 39.00 | 0.195 | 32.5 |
